# Supplementary material for: The role of social support in mitigating the effects of increased screen time on adolescent mental health
Source: PLOS Ment Health. 2025 Jan 6;2(1):e0000213. doi: 10.1371/journal.pmen.0000213 (PMC12798297; doi:10.1371/journal.pmen.0000213)
Supplement: S1 Table — (DOCX) [file pmen.0000213.s001.docx]

**S1 Table: Logistic regressions**

|  | Not depressed =1 if never felt depressed | | Life satisfaction score = 10 or 9 (the highest) | |
| --- | --- | --- | --- | --- |
|  | Or | p | Or | p |
| Screen time and social support | | | | |
| over 2h & no support | reference | | reference | |
| < 2h & support | 5.39 | <0.001 | 6.43 | <0.001 |
| over 2h & support | 2.71 | <0.001 | 3.01 | <0.001 |
| < 2h & no support | 2.01 | 0.03 | 1.22 | 0.54 |

Notes: Results showed that adolescents who received social support and had less than 2 hours of screen time had 5.39 times higher odds of never feeling depressed compared to those who spent over 2 hours on screen time and did not receive social support, holding all other variables constant. Similarly, adolescents receiving social support and less than 2 hours of screen time had 6.43 times higher odds of reporting a life satisfaction score of 9 or 10 (the highest scores) compared to those spending over 2 hours on screen time and not receiving social support.
