# Supplementary material for: The role of social support in mitigating the effects of increased screen time on adolescent mental health
Source: PLOS Ment Health. 2025 Jan 6;2(1):e0000213. doi: 10.1371/journal.pmen.0000213 (PMC12798297; doi:10.1371/journal.pmen.0000213)
Supplement: S2 Table — (DOCX) [file pmen.0000213.s002.docx]

**S2 Table: Availability of community support**

|  | Frequency of feeling depressed (1 daily; 5 never) | | Life satisfaction score on a (scale from 0 to 10) | |
| --- | --- | --- | --- | --- |
|  | Coefficient | p | Coefficient | p |
| Screen time and social support | | | | |
| over 2h & no community support available | reference | | reference | |
| < 2h & community support available | 0.33 | <0.001 | 0.64 | <0.001 |
| over 2h & community support available | 0.02 | 0.80 | 0.08 | 0.63 |
| < 2h & no community support available | 0.35 | 0.01 | 0.52 | 0.03 |
