# Supplementary material for: The role of social support in mitigating the effects of increased screen time on adolescent mental health
Source: PLOS Ment Health. 2025 Jan 6;2(1):e0000213. doi: 10.1371/journal.pmen.0000213 (PMC12798297; doi:10.1371/journal.pmen.0000213)
Supplement: Attachment — Submitted filename: response letter 0925.docx [file pmen.0000213.s003.docx]

Reviewer #1: Regarding the "background", improve the definition and delimitation of the variables: The term "social/emotional support" should be better defined. What does it mean in this context? What type of social support is being considered (family, peer, institutional or other)?

***RESPONSE****: We thank the reviewer for raising this comment. We agree that the independent variables should be more specific. In the revision, we described each variable and quoted the question that was asked to each respondent. For social support, the NHIS generally asked: "How often does [the adolescent] get the social and emotional support [the adolescent] needs? Would you say always, usually, sometimes, rarely, or never?" This is our key independent measure, indicating the actual receipt of support. Given the word limit of the abstract, we included the details in the data section.*

*This survey question is quite general. NHIS had a second measure to measure the availability of community support. The survey question was "Other than parents or adults living in [the adolescent]' home, is there at least one adult in [the adolescent] 's school, neighborhood, or community who makes a positive and meaningful difference in [the adolescent]' life? To our understanding, this question measured the availability of support. In this revision, we included the analysis of this measure of support availability in the appendix.*

*We acknowledge that the limitation of the measure and call for future research to better understand the specific social and emotional support that adolescents need.*

Specifying the temporal context: When you mention "during/after the COVID-19 pandemic", it is necessary to clearly delimit the phases you are referring to. Define whether we are talking about confinement, reopening, or both.

***RESPONSE****: We thank the reviewer for these comments. In our revision, we clarified that the interviews were conducted between January 1, 2022 to December 31, 2022, with responses reflecting this same time period. We included this detail in the paper. Since the U.S. federal government ended its COVID-19 Public Health Emergency declaration on May 11, 2023, we decided to consistently refer to the time period as during the pandemic.*

Objectives and approach of the study: The objective of the study is mentioned in a general way. It would be useful to specify what we are looking to measure, how screen time is expected to affect mental health, and how social support can moderate that relationship. Clarity and precision in the presentation of the method: Although the variables and techniques used are mentioned, important details are missing that increase the clarity and replicability of the study. For example, more information needs to be provided about sample selection, how data were recognized, and whether potentially confounding variables are controlled. Furthermore, it is not detailed how "social or emotional support" was measured.

***RESPONSE****: We appreciate the opportunity to include more details of our study objectives and approaches. In the introduction, we added two hypotheses following the study objective to make it more specific. In the method, we added details about the NHIS data and how we reached the final sample size and justified our model specifications. We also included the specific definitions of the measures.*

Further development in outcome measures: The description of the dependent variables is vague. You should explain what the "life satisfaction" measure is and how the "frequency of children who seem sad or depressed" is calculated. It is important that measurement tools are validated and accurately referenced to ensure scientific quality.

***RESPONSE****: We thank the reviewer for this comment and agree that we should clarify the measures. We did our best to describe the measures with as close phrasing to the interview questions as possible. The NHIS interview asked about the frequency of depression with the question: “How often does [the adolescent] seem very sad or depressed? Would you say: daily, weekly, monthly, a few times a year, or never?” And life satisfaction measure was captured from the question: “Using a scale of 0 to 10, where 0 means "very dissatisfied" and 10 means "very satisfied", how do you think [the adolescent] feels about [the adolescent] life as a whole these days?” We included the survey questions and restated our measures in the revision.*

Justification of the statistical analysis: Although it mentions ordinary least squares (OLS) regression, it does not explain why this technique was selected and whether possible violated assumptions (such as heteroscedasticity or multicollinearity) are considered. Additionally, it would be helpful to explain how the survey weights were performed and why they were necessary for this study.

***RESPONSE****: We selected OLS regression to estimate the linear association between screen time or social support and adolescent mental health. OLS was chosen because it enables us to capture the continuous and nuanced nature of these relationships. Nevertheless, we also conducted different sensitivity analyses using logit. Results were robust.*

*We used the Python packages Pandas and Statsmodels to run a linear regression and apply survey weights, referencing the NHIS built-in survey weight. We included this in the revision.*

Details of the population context: When indicating that the results are "nationally representative", it would be relevant to specify which country or region was taken into account and how this representativeness is ensured. National representativeness often requires a specific sampling design, which should be detailed.

***RESPONSE****: We added the U.S. population in the paper. As responded above, in the revision, we also talked about the sampling method in the paper. In our revision, we specified that the data is taken from the U.S. We also talked more about the sampling method in the paper. The Final Annual Weight should be used to generate national estimates based on the NHIS. This weight accounts for sampling probabilities and nonresponse. We used “WTFA_C” for our weight variables.*

Reviewer #2: I’ve read the work titled “The Role of Social Support in Mitigating the Effects of Increased Screen Time on Adolescent Mental Health”. The introduction is well written and summarizes the current state of the art well. The literature used is appropriate and recent.
The methods are generally well written. Nevertheless, I would add more details to some passages in order to make this part more informative. I recommend adding more information regarding the population from which the study sample was drawn and adding more details regarding the cohort selection. It might be helpful to add a flow chart illustrating the cohort selection procedure. Having this information would make it easier for the study to be replicated. In any case, I think the methods section is a bit lacking. I recommend reviewing this part better.

***RESPONSE****: We appreciate the reviewer’s great comments that helped us improve the clarity of our study. In the revision we added more details to the methods. Specifically, we added the introduction data, the justification of the model specification, the approach, more details on the measures. We agree that we should clarify the sample. In the revision, we added a new table of sample characteristics.*

*We want to note that we did not select the sample. NHIS is a nationally representative survey. The total of 2022 sample child include 2,758 adolescents aged 12-17. Among them 2,649 had full records. And we applied the survey weight. In the revision, we talked more about the sampling method in the paper. The Final Annual Weight should be used to generate national estimates based on the NHIS. This weight accounts for sampling probabilities and nonresponse.* *We used “WTFA_C” for our weight variables.*

In the Results section, where appropriate, in the various titles of the tables I recommend adding more information such as, for example, the time period to which the data refers.

***RESPONSE****: We clarify that interviews were conducted between January 1, 2022 to December 31, 2022, with responses reflecting this same time period. We included this detail in the paper.*

The discussion is generally well written. Particularly, I appreciated the section on limitations of the study. Nonetheless, I recommend discussing the weaknesses in more detail (e.g. adding any bias related to the data source). Finally, I recommend including a series of strengths related to the data source or study design that was used in order to give greater value to the work.

***RESPONSE****: We thank the reviewer for the comment. In the revision, we have added more details regarding the study's limitations. We emphasized that we conducted a cross-sectional analysis, and therefore, the results cannot infer causality. In addition, we would like to note that the NHIS is a nationally representative dataset that has been widely adopted to examine health outcomes and healthcare utilization. Lastly, we appreciate the reviewer’s suggestion to add a section on the study's strengths, which we have now included in the revised manuscript.*
