# Supplementary material for: The role of social support in mitigating the effects of increased screen time on adolescent mental health
Source: PLOS Ment Health. 2025 Jan 6;2(1):e0000213. doi: 10.1371/journal.pmen.0000213 (PMC12798297; doi:10.1371/journal.pmen.0000213)
Supplement: Attachment — Submitted filename: response letter.docx [file pmen.0000213.s004.docx]

PMEN-D-24-00336R1
The Role of Social Support in Mitigating the Effects of Increased Screen Time on Adolescent Mental Health
PLOS Mental Health

**RESPONSE LETTER**:
**Reviewer** #1: Specify the statistical aspect of the research work, if the study had the objective of estimating the association between Screen time, social/emotional support, and mental health among adolescents. what is the level during/after the COVID-19 pandemic. The Conclusion is clear, the existence of an association between the study variables was determined. BUT IT WOULD BE IMPORTANT TO SPECIFY THE RESULTS (the level, degree or strength of the association).

**RESPONSE**: We thank the reviewer for their thoughtful comments. In this revision, we have updated the discussion section by adding the following: “Specifically, adolescents who reported less than 2 hours of screen time and received social support experienced a 0.79-point reduction in depression frequency and a 1.34-point increase in life satisfaction.”

In the main results, we used OLS estimation to capture the continuous and granular nature of these relationships. Our findings are consistent with the logistic regression results included in the appendix. To provide a comprehensive understanding, we also included interpretations of the logistic regression findings in the appendix: “Results showed that adolescents who received social support and had less than 2 hours of screen time had 5.39 times higher odds of never feeling depressed compared to those who spent over 2 hours on screen time and did not receive social support, holding all other variables constant. Similarly, adolescents receiving social support and less than 2 hours of screen time had 6.43 times higher odds of reporting a life satisfaction score of 9 or 10 (the highest scores) compared to those spending over 2 hours on screen time and not receiving social support.”

We hope these revisions address the reviewer’s concerns and provide clarity on the consistency and interpretation of our findings.

**Reviewer #2**: I have read the article titled “The Role of Social Support in Mitigating the Effects of Increased Screen Time on Adolescent Mental Health” and am pleased that the authors have addressed the issues raised in the previous round. Currently, the manuscript is a well-written research paper whose objective is to identify profiles of prompt, adequate and continuous physician follow-up care and other physician practice features, and to estimate the association between screen time, social/emotional support, and mental health among adolescents during/post the COVID-19 pandemic. I believe the manuscript meets the publication standards of the journal.

**RESPONSE:** We thank the reviewer and appreciate the comments to make our study more robust.
