# Supplementary material for: The role of social support in mitigating the effects of increased screen time on adolescent mental health
Source: PLOS Ment Health. 2025 Jan 6;2(1):e0000213. doi: 10.1371/journal.pmen.0000213 (PMC12798297; doi:10.1371/journal.pmen.0000213)
Supplement: Attachment — Submitted filename: response letter 1128 (1).docx [file pmen.0000213.s005.docx]

***Editor’s Comment to Authors:***

*The discussion section should be expanded to include a more detailed examination of the relationship between excessive screen time and its impact on mental health, particularly depression and overall health quality. For example, excessive screen time has been linked to negative outcomes such as poor sleep quality, longer sleep onset latency, delayed bedtimes, shorter sleep duration, and increased daytime fatigue in school-aged children and adolescents. Additionally, evidence suggests that increased screen use may contribute to deteriorating relationships with parents and peers and is often employed as a mechanism for self-regulation, emotional escape, or stress relief.*

*Incorporating these points into your discussion will strengthen the manuscript by situating your findings within the broader literature on screen time and its effects on mental and physical well-being*."

**RESPONSE**: We greatly appreciate the Editor’s careful review and insightful comments. We agree that adding more discussion can strengthen the manuscript. Accordingly, we have revised the manuscript and added the following:

Excessive screen time during adolescence has been linked to a variety of negative health outcomes. Studies have found that high screen time is associated with psychosocial problems in adolescence, such as increased depressive symptoms and anxiety, as well as a higher likelihood of being overweight.^18^ Moreover, excessive screen time has been shown to disrupt sleep patterns, contributing to shorter sleep duration, delayed sleep onset, and more frequent sleep disturbances.^19,20^ Screen time is also linked to poor attachment to parents and peers, which may delay social and emotional development.^21^ Research further indicates that excessive screen use can negatively impact academic performance due to reduced focus and increased procrastination.^22^ Additionally, prolonged screen exposure has been associated with reduced physical activity, leading to a sedentary lifestyle and increased risk of metabolic disorders.^23^ These findings collectively demonstrate that excessive screen time poses significant risks to well-being, particularly during the critical developmental stage of adolescence.^24^

18. Busch V, Manders LA, de Leeuw JR. Screen time associated with health behaviors and outcomes in adolescents. Am J Health Behav. 2013 Nov;37(6):819-30. doi: 10.5993/AJHB.37.6.11. PMID: 24001631.
19. Hale L, Guan S. Screen time and sleep among school-aged children and adolescents: a systematic literature review. Sleep Med Rev. 2015 Jun;21:50-8. doi: 10.1016/j.smrv.2014.07.007. Epub 2014 Aug 12. PMID: 25193149; PMCID: PMC4437561.
20. Ahmed GK, Abdalla AA, Mohamed AM, Mohamed LA, Shamaa HA. Relationship between time spent playing internet gaming apps and behavioral problems, sleep problems, alexithymia, and emotion dysregulations in children: a multicentre study. Child Adolesc Psychiatry Ment Health. 2022 Aug 16;16(1):67. doi: 10.1186/s13034-022-00502-w. PMID: 35974366; PMCID: PMC9380675.
21. Richards R, McGee R, Williams SM, Welch D, Hancox RJ. Adolescent Screen Time and Attachment to Parents and Peers. *Arch Pediatr Adolesc Med.* 2010;164(3):258–262. doi:10.1001/archpediatrics.2009.280
22. Madigan, S., et al. (2019). Associations Between Screen Use and Child Language Skills: A Systematic Review and Meta-analysis. *JAMA Pediatrics*, 173(3), 244–250. [https://jamanetwork.com/journals/jamapediatrics/fullarticle/2722666]
23. Boone, J. E., et al. (2007). Screen time and physical activity during adolescence: longitudinal effects on obesity in young adulthood. *International Journal of Behavioral Nutrition and Physical Activity*, 4, 26. [<https://ijbnpa.biomedcentral.com/articles/10.1186/1479-5868-4-26>]
24. Twenge, J. M., & Campbell, W. K. (2018). Associations Between Screen Time and Lower Psychological Well-Being Among Children and Adolescents: Evidence From a Population-Based Study. *Preventive Medicine Reports*, 12, 271–283.
